# Usefulness of a Low-Dose Sclerosing Agent for the Treatment of Vascular Lesions in the Tongue

**DOI:** 10.7759/cureus.45323

**Published:** 2023-09-15

**Authors:** Taeil Lim, Doogyum Kim, Hyun-Woo Lee, Joo-Young Ohe, Junho Jung

**Affiliations:** 1 Oral and Maxillofacial Surgery, Kyung Hee University, Seoul, KOR; 2 Oral and Maxillofacial Surgery, Eulji University, Uijeongbu, KOR

**Keywords:** sts, sodium tetradecyl sulfate, sclerotherapy, hemangioma, vascular malformation

## Abstract

Hemangiomas and vascular malformations are common benign lesions of vessels in the cervical region. However, the lesions may not completely disappear and may require surgical or nonsurgical intervention. Several treatment options, including surgical excision, steroid injection, laser therapy, and sclerotherapy, are available. Surgical excision is a commonly used treatment; however, in cases of hemangiomas of the tongue, excision of the lesion may cause esthetic or functional impairments, including speech and swallowing. Sclerotherapy is a simple and safe method for treating vascular lesions conservatively. In this case report, two patients with a vascular lesion of the tongue underwent conservative sclerotherapy without surgical excision using a sclerosing agent (sodium tetradecyl sulfate). Both patients showed regression of the lesion without complications. As presented in these cases, repeated injections of low-dose 1% sodium tetradecyl sulfate as a sclerosing agent were safe and showed satisfactory outcomes.

## Introduction

Hemangiomas are benign tumors with vascular anomalies that show rapid growth with endothelial cell proliferation in the proliferative phase, followed by slow involution, and 90% of hemangiomas are completely involuted before nine years of age. In contrast, vascular malformations demonstrate a normal endothelial cell cycle with no hyperplastic cells exhibit gradual growth and rare regression. Vascular malformations manifest from birth and exhibit a slow expansion over the course of a lifetime [1-4].

The most common benign vascular tumors are infantile hemangiomas, which are not visible at birth but appear within a few weeks. Congenital hemangiomas appear similar to infantile hemangiomas but are fully developed at birth. Rapidly involuting congenital hemangiomas typically undergo complete involution by the age of two. However, they continue to grow proportionally during childhood and do not undergo involution [5].

For the diagnosis of a vascular lesion, palpation or a diascopy test of the lesion shows the disappearance of the blood in the lesion, and this test can make a differential diagnosis of vascular lesions from other benign tumors such as fibroma or mucocele [6].

Several methods have been introduced for the treatment of vascular lesions, including surgical excision, laser treatment, electrocoagulation, cryotherapy, and sclerotherapy. General condition, age, and location are important factors in selecting a treatment method, and sclerotherapy is used for lesions where surgical intervention may cause esthetic or functional impairment [1,7,8]. Frequent complications after sclerotherapy include ulceration, pain, swelling, local inflammation, and injection site bruising [9,10].

In this case report, we describe two patients with vascular lesions of the tongue who underwent sclerotherapy with successful reduction of the lesion.

## Case presentation

Case 1

A 66-year-old man with swelling and pain on the right side of the tongue visited the Department of Oral and Maxillofacial Surgery at Kyung Hee University Medical Center (Seoul, Republic of Korea). The patient reported no relevant medical history and was uncertain about the timing of the occurrence of the swelling.

**Figure 1:**
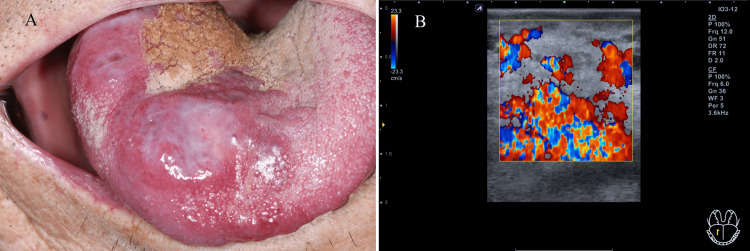
Vascular lesion on the anterior region of the tongue (A) Clinical appearance before treatment; (B) Ultrasonography image before treatment

On clinical examination, a bluish, oval, dome-shaped swelling measuring 2.0 cm in diameter was observed on the dorsal and ventral sides of the tongue, which was painful during functioning and chewing. In addition, the patient complained of an unclear pronunciation. Doppler ultrasonography revealed a hyperechoic lesion with large blood vessels. Color Doppler images of the region revealed enlarged vessels with a high density and irregular blood-filled states within the lesion (Figure 1).

**Figure 2:**
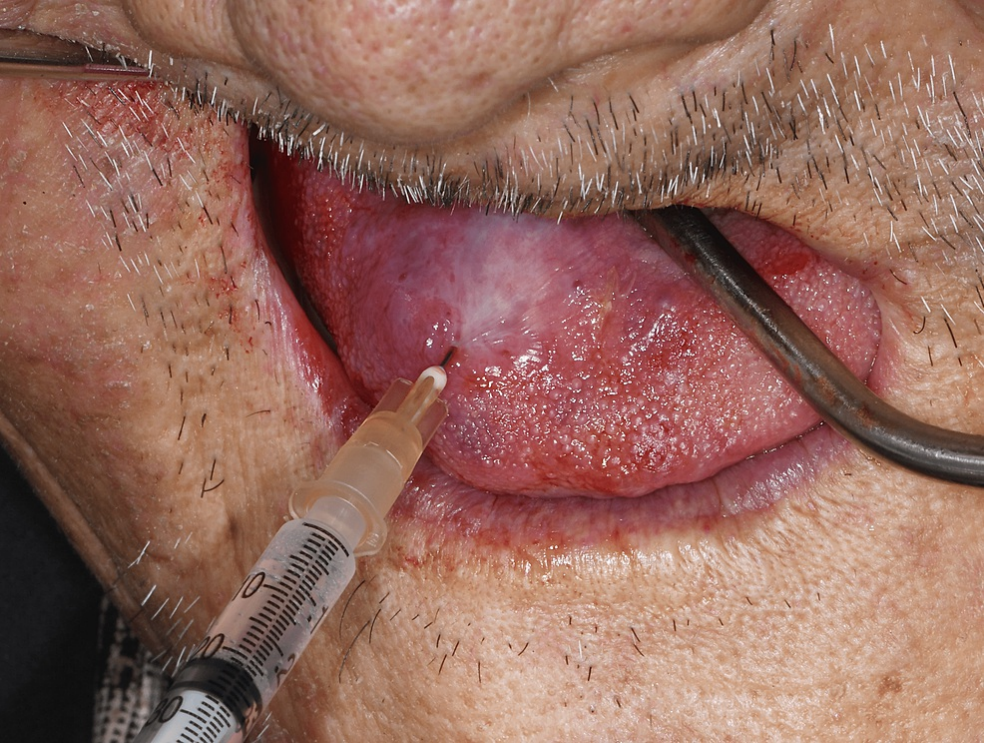
Injection of sclerosing agent (1% sodium tetradecyl sulfate)

Considering its large size and location, conservative treatment using sclerotherapy (1% sodium tetradecyl sulfate [STS]) was preferred, as surgical excision of the lesion may cause massive bleeding and esthetic or functional impairment. Approximately 2 mL of the sclerosing agent (1% STS) was injected at the center and border of the lesion. The lesion was compressed for 10-15 minutes after direct injection of 1% STS. The patient was asked to report every two weeks (Figure 2).

**Figure 3:**
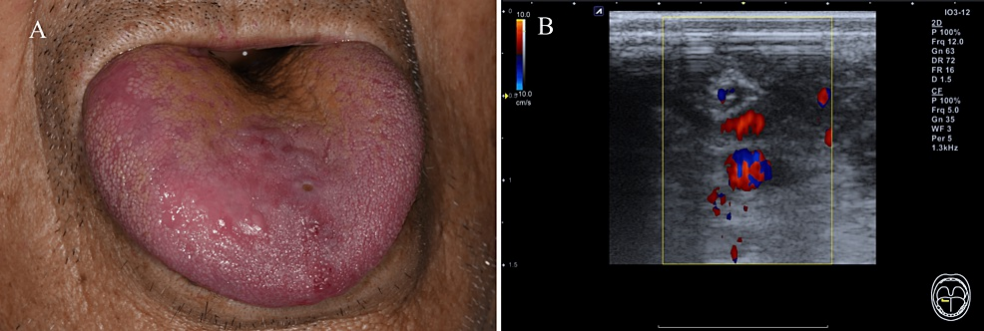
Vascular lesion after sclerotherapy (A) seven-month follow-up appearance (B) four-month follow-up ultrasonography doppler image, which shows remarkably decreased size and flow of the lesion.

At the four-month follow-up, both a reduction in the size of the lesion and a decrease in blood flow were confirmed in comparison to the previous ultrasonographic findings (Figure 3). The patient showed a substantial decrease in lesion size with improved esthetics and speech function during the seven-month follow-up (Figure 3).

Case 2

A 28-year-old woman with bluish, dome-shaped lesions on the anterior and posterior tongue visited the Department of Oral and Maxillofacial Surgery at Kyung Hee University Medical Center. The patient had noticed the lesion two years earlier and had no relevant medical history.

**Figure 4:**
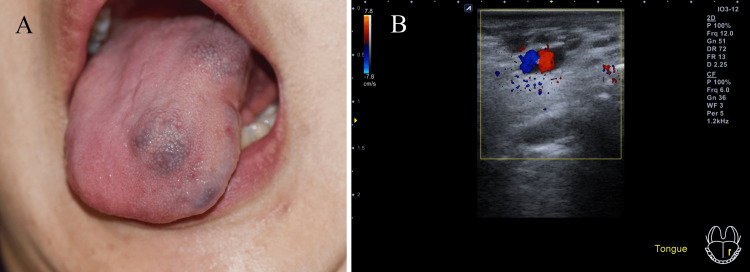
Vascular lesion on the anterior region of the tongue before sclerotherapy (A) Clinical appearance; (B) Ultrasonography doppler image

Clinical examination showed purplish dome-shaped swelling on the anterior side, border, and posterior area of the dorsal surface of the tongue. The size of the anterior and posterior lesions measured 1.0 cm in diameter. The patient complained of frequent chewing around the lesion, and no other related symptoms were observed. Ultrasonography revealed a mass extending from the middle to the tip of the left tongue. Margins with multiple hypoechoic spaces below the surface were clear, and no erosive or infiltrative findings were observed. Blood circulation was observed in the lesions on the anterior area of the tongue during Doppler ultrasonography phase selection but not in the entire cavity (Figure 4). 

**Figure 5:**
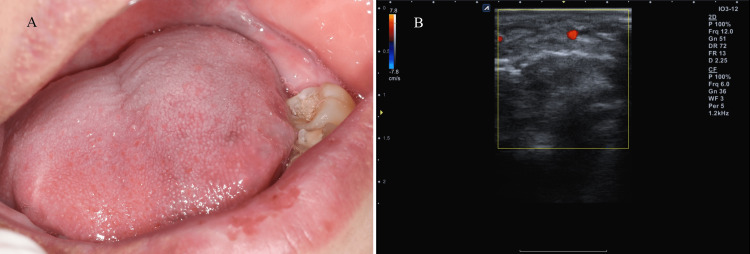
Three-month follow-up after sclerotherapy (A) Clinical appearance (B) Ultrasonography doppler image

After identifying the location and vascular characteristics of the lesion, 2 mL of 1% STS was injected into the center and border of the lesion, and the lesion was compressed for approximately 10-15 minutes to achieve stasis. During each visit, approximately 2 mL of STS was administered at two-week intervals, resulting in a total treatment period of three months with six injections. The lesions showed satisfactory involution at the three-month follow-up. The size of the lesion regressed considerably compared with its initial state (Figure 5).

## Discussion

Sclerotherapy offers distinct advantages in the treatment of intraoral vascular lesions. Unlike surgical excision, sclerotherapy provides a non-invasive alternative. In these specific cases, the size of vascular lesions was significantly reduced without encountering any complications.

Hemangiomas and vascular malformations are common tumors in infancy, and more than 60% of all hemangiomas occur in the head and neck regions. According to the Mulliken and Glowacki classification, vascular lesions can be divided into hemangiomas and vascular malformations [4]. Hemangiomas exhibit endothelial cell proliferation, rapid growth, and gradual involution. Vascular malformations have a normal endothelial cell cycle, a normal growth rate and persist throughout life. Vascular malformations do not contain hyperplastic cells; instead they comprise dilated vessels with distinctive vascular architectures, encompassing veins, lymphatic vessels, venules, capillaries, arteries, or a combination of vessel types [1,3]. The differential diagnosis of hemangioma and vascular malformation requires a biopsy, but since the treatment methods for these two diseases are similar, biopsies were not performed in both cases.

Most hemangiomas and vascular malformations do not require treatment. Approximately 90% of hemangiomas regress during the involuting phase before nine years of age [2-4,11]. However, the lesion may not completely disappear and may require surgical or non-surgical intervention.

Surgical or conservative treatment was used in cases of ulceration, pain, bleeding, airway obstruction, or esthetic impairment. Corticosteroids can be administered via both systemic and intralesional injections [12]. However, steroids are effective only during the proliferative phase, and there is a potential for serious side effects [11]. Surgical excision can be performed in cases of uncontrolled ulceration and bleeding or when the lesion does not respond to nonsurgical treatments. However, surgical excision is limited when massive bleeding or a crucial organ injury is anticipated during surgery [12-14]. Sclerotherapy can be considered an alternative to surgical excision. Sclerotherapy is a simple and minimally invasive method that can effectively reduce the size of the vascular lesion [1,13]. In this study, sclerotherapy was chosen because it is a simple and inexpensive method.

There are three types of sclerosing agents: detergents, hypertonic solutions, and chemical irritants. All these agents cause endothelial cellular injury, followed by sclerosis of the vessel lesion [9]. STS, polidocanol, and ethanolamine oleate belong to the detergent category. Long fatty chains of detergents form micelles in the lesion, which remove endothelial cell surface proteins from the cell. Denaturation of endothelial cell proteins causes cell damage and fibrosis in target vessels [9,15].

Sclerotherapy is a comparatively simple and safe method; however, its limitations must be considered. Contraindications to sclerotherapy include allergic sensitivity to sclerosants, severe systemic diseases such as uncontrolled diabetes, and local lesion infections. In addition, bleeding disorders, pregnancy, and a history of deep vein thrombosis should be carefully considered [9,15]. Commonly reported side effects of using STS in sclerotherapy include pain, swelling, ulceration, fever, and hyper- or hypopigmentation [10,16,17].

Sclerotherapy with 3% STS has shown favorable outcomes for the treatment of oral vascular lesions. In a study by Agarwal et al., including 20 patients, 95% of the patients with hemangiomas on the tongue, lip, and palate showed complete regression using 3% STS, and partial regression was achieved in one patient. In a study by Minkow et al., including 24 patients with hemangiomas of the lips, tongue, palate, and cheek mucosa treated with 3% STS, complete regression was achieved after a single injection in small tumors (0-1.5 cm), whereas large tumors (2-4 cm) required up to 10 STS injections. No scarring or defects were observed [10,16,17].

A study conducted by Gorman et al., involving sclerotherapy treatment for 34 patients with venous malformation, revealed a 30% recurrence rate when 3% STS was used. On average, these recurrences occurred 21.5 months after the last injection [18]. This study suggests that a sufficient follow-up period is necessary because there is a possibility of recurrence even after the lesion has completely disappeared through sclerotherapy.

Similar to other sclerosing agents, STS may cause pain, ulceration, edema, and anaphylaxis. Although 3% STS is commonly used, Choi et al. reported three cases in which 1% STS was employed to mitigate side effects [13]. In our cases, 1% STS was injected to reduce these complications [13].

## Conclusions

Sclerotherapy offers distinct advantages in the treatment of intraoral vascular lesions. Unlike surgical excision, which can result in bleeding during the procedure and postoperative scarring, sclerotherapy provides a non-invasive alternative. When anticipating challenges in controlling intraoperative bleeding or being concerned about potential esthetic issues post-surgery, opting for nonsurgical approaches should be considered. In this specific case, the vascular lesion size was significantly reduced without encountering any complications, obviating the need for additional surgical interventions. Consequently, it becomes evident that sclerotherapy holds the potential to yield superior esthetic outcomes in the management of vascular lesions.
